# Association of Preoperative Frailty and Operative Stress With Mortality After Elective vs Emergency Surgery

**DOI:** 10.1001/jamanetworkopen.2020.10358

**Published:** 2020-07-13

**Authors:** Myrick C. Shinall, Ada Youk, Nader N. Massarweh, Paula K. Shireman, Shipra Arya, Elizabeth L. George, Daniel E. Hall

**Affiliations:** 1 Department of Surgery, Vanderbilt University Medical Center, Nashville, Tennessee; 2 Center for Health Equity Research and Promotion, VA Pittsburgh Healthcare System, Pittsburgh, Pennsylvania; 3 Department of Biostatistics, Graduate School of Public Health, University of Pittsburgh, Pittsburgh, Pennsylvania; 4 Center for Innovations in Quality, Effectiveness and Safety, Michael E. DeBakey VA Medical Center, Houston, Texas; 5 Michael E. DeBakey Department of Surgery, Baylor College of Medicine, Houston, Texas; 6 Department of Surgery, University of Texas Health San Antonio, San Antonio; 7 South Texas Veterans Health Care System, San Antonio; 8 Division of Vascular Surgery, Stanford University School of Medicine, Stanford, California; 9 Surgical Service Line, VA Palo Alto Healthcare System, Palo Alto, California; 10 Division of Health Services Research and Development, VA Palo Alto Healthcare System, Palo Alto, California; 11 Department of Surgery, University of Pittsburgh, Pittsburgh, Pennsylvania; 12 Wolff Center, University of Pittsburgh Medical Center, Pittsburgh, Pennsylvania

## Abstract

This cohort study investigates whether preoperative patient frailty and operative stress are associated with postoperative mortality for patients undergoing elective vs emergent surgical procedures.

## Introduction

Our group recently examined the associations among preoperative frailty, operative stress, and postoperative mortality in a retrospective cohort published in *JAMA Surgery*.^1^ Frail and very frail patients had high mortality rates at 30, 90, and 180 days even after low-stress operations, a finding that triggered questions about whether inclusion of patients undergoing emergency operations may have been associated with the high mortality rate. We hypothesized that postoperative mortality would increase with increasing frailty and increasing operative stress for both elective and emergent operations, with high levels of mortality for frail and very frail patients even after low-stress, elective operations.

## Methods

The VA Pittsburgh Healthcare System institutional review board determined this analysis to be exempt because data were deidentified; thus, no consent was needed. This study follows the Strengthening the Reporting of Observational Studies in Epidemiology (STROBE) reporting guideline.

This cohort study used data from the Veterans Affairs Surgical Quality Improvement Program for noncardiac surgical procedures performed between April 1, 2010, and March 31, 2014, for veterans with available 1-year postoperative vital status. Exposures of interest were urgency (emergent vs elective), frailty (measured by the Risk Analysis Index [RAI]), and operative stress (measured by the Operative Stress Score [OSS]). Operative urgency was defined by the binary Veterans Affairs Surgical Quality Improvement Program variable for emergent operations. The RAI is based on the accumulation of deficits model of frailty and uses demographic factors (including age), comorbidities, cognitive decline, residence in a facility, and activities of daily living to quantify frailty, with higher scores indicating greater frailty (eFigure in the Supplement).^2,3,4,5^ The OSS was developed using modified Delphi consensus methods to rate the 565 most common *Current Procedural Terminology* codes included in Veterans Affairs Surgical Quality Improvement Program on a scale of 1 to 5 by degree of physiologic stress experienced by patients, with higher scores indicating more stress (eTable in the Supplement).^1^ Patients were categorized as robust, normal, frail, and very fail by RAI score (RAI ≤20, 21-29, 30-39, and ≥40, respectively).^1,3^ The outcomes were mortality at 30, 90, and 180 days. *P* values were calculated at the 95% significance level. The χ^2^ test for trend was used to test for increasing mortality with increasing OSS level and frailty. All analyses were performed using STATA statistical software version 14 (StataCorp). Data analysis was performed from January 2020 to May 2020.

## Results

The data set included 432 828 patients (mean [SD] age, 61.0 [12.9] years; 401 453 male [92.8%]; 299 809 white [69.3%]), with a mean (SD) RAI score of 21.25 (7.34) (further demographic data are available in the original article).^1^ There were 21 748 (5.02%) emergent and 411 080 (94.98%) elective procedures. Emergent procedures constituted 7308 (3.66%), 7588 (4.05%), 4519 (12.35%), and 2333 (25.60%) of all procedures for robust, normal, frail, and very frail patients, respectively.

Although mortality after emergent operations was higher than that after elective operations, frail and very frail patients experienced significantly higher mortality than their more robust counterparts even after elective surgery (Table). For example, after the lowest-stress elective operations (OSS 1), 180-day mortality rates for frail and very frail patients were more than 10 times the rate among robust patients (9.14% [95% CI, 8.27%-10.06%] and 33.33% [95% CI, 28.745-38.18%] vs 0.52% [95% CI, 0.42%-0.63%], respectively). Findings were similar for the highest-stress elective operations (OSS 5), with 180-day mortality rates for frail and very frail patients of 19.01% (95% CI, 15.31%-23.18%) and 14.29% (95% CI, 9.17%-20.83%), respectively, compared with only 5.88% (95% CI, 4.06%-8.20%) for robust patients. The 30- and 90-day mortality rates (Figure) were also markedly increased among frail and very frail patients after elective operations with rates that mirror the mortality seen in the overall cohort including emergent operations.

**Table.** Mortality at 180 Days Postoperatively by Level of Operative Stress

Frailty stratum and operative urgency	Mortality rate, mean (95% CI), %					*P* value for OSS^a^
	OSS 1	OSS 2	OSS 3	OSS 4	OSS 5	
Robust (RAI ≤20)						
All (n = 199 677)	0.54 (0.44-0.65)	0.43 (0.39-0.46)	1.03 (0.94-1.12)	2.69 (2.37-3.04)	5.93 (4.23-8.05)	<.001
Elective (n = 192 369)	0.52 (0.42-0.63)	0.39 (0.36-0.43)	0.91 (0.83-1.00)	2.59 (2.27-2.95)	5.88 (4.06-8.20)	<.001
Emergent (n = 7308)	1.20 (0.44-2.59)	1.56 (1.18-2.02)	3.17 (2.52-3.92)	4.37 (2.76-6.55)	6.19 (2.30-12.98)	<.001
Normal (RAI 21-29)						
All (n = 187 459)	1.97 (1.78-2.17)	1.45 (1.37-1.53)	3.29 (3.15-3.44)	5.78 (5.41-6.18)	8.61 (7.29-10.08)	<.001
Elective (n = 179 871)	1.90 (1.72-2.10)	1.35 (1.27-1.42)	2.87 (2.73-3.01)	5.31 (4.94-5.70)	8.26 (6.91-9.76)	<.001
Emergent (n = 7588)	5.41 (3.42-8.07)	5.24 (4.38-6.21)	9.45 (8.54-10.42)	13.05 (10.90-15.46)	12.90 (7.56-20.11)	<.001
Frail (RAI 30-39)						
All (n = 36 579)^b^	9.40 (8.54-10.32)	9.19 (8.68-9.72)	16.22 (15.65-16.80)	18.03 (16.83-19.28)	18.43 (15.04-22.23)	<.001
Elective (n = 32 060)^b^	9.14 (8.27-10.06)	8.45 (7.93-8.90)	14.85 (14.25-15.46)	14.88 (13.65-16.18)	19.01 (15.31-23.18)	<.001
Emergent (n = 4519)^b^	15.70 (10.61-22.01)	17.74 (15.36-20.33)	23.41 (21.78-25.09)	31.04 (27.76-34.47)	14.93 (7.40-25.74)	<.001
Very frail (RAI ≥40)						
All (n = 9113)^b^	34.91 (30.58-39.44)	31.92 (29.68-34.21)	43.00 (41.69-44.32)	41.97 (39.23-44.75)	17.10 (12.07-23.17)	.001
Elective (n = 6780)^b^	33.33 (28.74-38.18)	29.15 (26.76-31.64)	40.20 (38.69-41.73)	34.10 (30.78-37.55)	14.29 (9.17-20.83)	.24
Emergent (n = 2333)^b^	45.16 (32.48-58.32)	44.63 (38.90-50.47)	50.82 (48.22-53.42)	54.81 (50.23-59.34)	28.21 (15.00-44.87)^c^	.11

Abbreviations: OSS, Operative Stress Score; and RAI, Risk Analysis Index.

^a^ χ^2^ test for trend.

^b^ All *P* values for trend across frailty are <.001 except where noted otherwise (χ^2^ test for trend).

^c^ *P* = .001 (χ^2^ test for trend).

**Figure.**
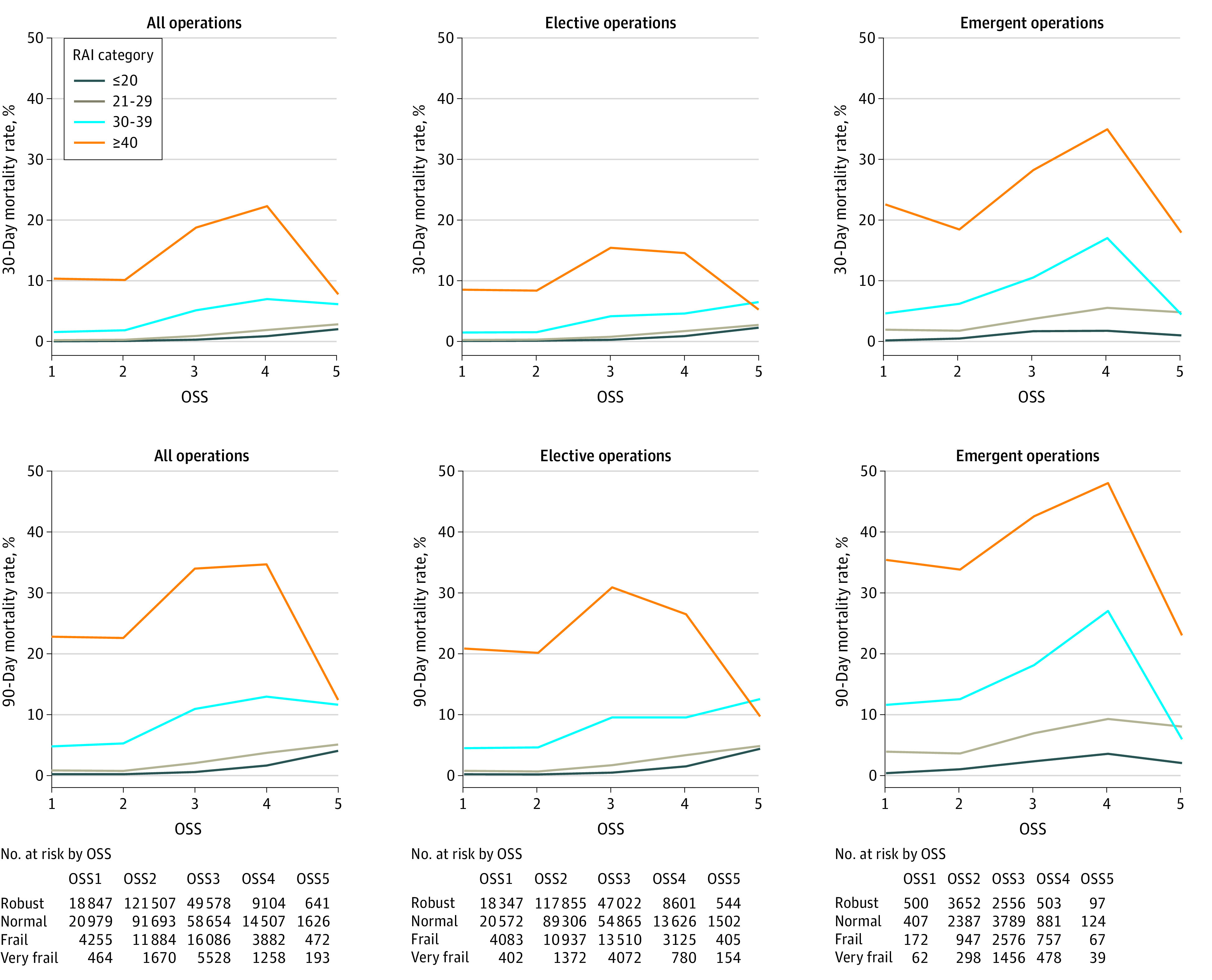
Mortality Rate at 30 and 90 Days After Surgery OSS indicates Operative Stress Score and RAI, Risk Analysis Index.

## Discussion

Frail and very frail patients have substantial postoperative mortality after elective operations of any level of operative stress, with even higher rates of mortality after emergent operations. The previously demonstrated high levels of postoperative mortality for frail and very frail patients did not result solely from including emergent operations in the overall cohort. For both emergent and elective operations, mortality had a complex, nonlinear association with operative stress, suggesting selection effects by operating surgeons, especially with higher OSS and RAI levels. Frailty remains a primary factor associated with postoperative outcomes after elective operations. This study is limited by its retrospective nature and the use of the Veterans Affairs Surgical Quality Improvement Program data set, which may not represent the nonveteran population. Nevertheless, these results reinforce the need for frailty assessment at the point of care to achieve 4 important goals: (1) to risk-stratify patients for operations, especially operations perceived as being routine or low risk; (2) to help physicians and frail patients in both elective or emergent settings make informed decisions about surgical options, including palliative approaches; (3) to optimize care for these patients preoperatively whenever possible; and (4) to provide goal-concordant care, which may involve choosing to operate even when the risk of mortality is high if substantial improvements in quality of life are expected.

## Notes

Supplement. **eTable.** OSS Scores for 565 *CPT* Codes **eFigure.** Revised RAI Scoring **eReferences.**
