# Supplementary material for: Association of Preoperative Frailty and Operative Stress With Mortality After Elective vs Emergency Surgery
Source: JAMA Netw Open. 2020 Jul 13;3(7):e2010358. doi: 10.1001/jamanetworkopen.2020.10358 (PMC7358909; doi:10.1001/jamanetworkopen.2020.10358)
Supplement: Supplement. — eTable. OSS Scores for 565 CPT Codes eFigure. Revised RAI Scoring eReferences. [file jamanetwopen-3-e2010358-s001.pdf]

Supplementary Online Content

Shinall MC Jr, Youk A, Massarweh NN, et al. Association of preoperative frailty and operative stress with mortality after elective vs emergency surgery. *JAMA Netw Open.* 2020;3(7):e2010358. doi:10.1001/jamanetworkopen.2020.10358

eTable. OSS Scores for 565 *CPT* Codes
eFigure. Revised RAI Scoring
eReferences

This supplementary material has been provided by the authors to give readers additional information about their work.

© 2020 Shinall MC Jr et al. *JAMA Network Open.*

eTable: OSS Scores for 565 *CPT* Codes

CPT Code	OSS	CPT Description
10121	1	Incision and removal of foreign body, subcutaneous tissues; complicated
10140	1	Incision and drainage of hematoma, seroma or fluid collection
10180	2	Incision and drainage, complex, postoperative wound infection
11004	3	Debridement of skin, subcutaneous tissue, muscle and fascia for necrotizing soft tissue infection; external genitalia and perineum
11005	3	Necrotizing soft tissue infection; abdominal wall, with or without fascial closure
11006	3	Debridement of skin, subcutaneous tissue, muscle and fascia for necrotizing soft tissue infection; external genitalia, perineum and abdominal wall, with or without fascial closure
11042	2	Debridement, subcutaneous tissue (includes epidermis and dermis, if performed); first 20 sq cm or less
11043	2	Debridement, muscle and/or fascia (includes epidermis, dermis, and subcutaneous tissue, if performed); first 20 sq cm or less
11044	2	Debridement, bone (includes epidermis, dermis, subcutaneous tissue, muscle and/or fascia, if performed); first 20 sq cm or less
14060	1	Adjacent tissue transfer or rearrangement, eyelids, nose, ears and/or lips; defect 10 sq cm or less
15100	2	Split-thickness autograft, trunk, arms, legs; first 100 sq cm or less, or 1% of body area of infants and children (except 15050)
15120	2	Split-thickness autograft, face, scalp, eyelids, mouth, neck, ears, orbits, genitalia, hands, feet, and/or multiple digits; first 100 sq cm or less, or 1% of body area of infants and children (except 15050)
15260	1	Full thickness graft, free, including direct closure of donor site, nose, ears, eyelids, and/or lips; 20 sq cm or less
15576	2	Formation of direct or tubed pedicle, with or without transfer; eyelids, nose, ears, lips, or intraoral
15620	2	Delay of flap or sectioning of flap (division and inset); at forehead, cheeks, chin, neck, axillae, genitalia, hands, or feet
15630	2	Delay of flap or sectioning of flap (division and inset); at eyelids, nose, ears, or lips
15731	2	Forehead flap with preservation of vascular pedicle (eg, axial pattern flap, paramedian forehead flap)
15732	3	Muscle, myocutaneous, or fasciocutaneous flap; head and neck (eg, temporalis, masseter muscle, sternocleidomastoid, levator scapulae)
15734	3	Muscle, myocutaneous, or fasciocutaneous flap; trunk
15738	3	Muscle, myocutaneous, or fasciocutaneous flap; lower extremity
15830	2	Excision, excessive skin and subcutaneous tissue (includes lipectomy); abdomen, infraumbilical panniculectomy
15877	2	Suction assisted lipectomy; trunk
15940	2	Excision, ischial pressure ulcer, with primary suture
15946	2	Excision, ischial pressure ulcer, with ostectomy, in preparation for muscle or myocutaneous flap or skin graft closure
17311	2	Mohs micrographic technique, including removal of all gross tumor, surgical excision of tissue specimens, mapping, color coding of specimens, microscopic examination of specimens by the surgeon, and histopathologic preparation including routine stain(s) (eg, hematoxylin and eosin, toluidine blue), head, neck, hands, feet, genitalia, or any location with surgery directly involving muscle, cartilage, bone, tendon, major nerves, or vessels; first stage, up to 5 tissue blocks
19020	2	Mastotomy with exploration or drainage of abscess, deep
19120	2	Excision of cyst, fibroadenoma, or other benign or malignant tumor, aberrant breast tissue, duct lesion, nipple or areolar lesion (except 19300), open, male or female, 1 or more lesions

© 2020 Shinall MC Jr et al. *JAMA Network Open.*

CPT Code	OSS	CPT Description
19125	2	Excision of breast lesion identified by preoperative placement of radiological marker, open; single lesion
19300	2	Mastectomy for gynecomastia
19301	2	Mastectomy, partial (eg, lumpectomy, tylectomy, quadrantectomy, segmentectomy);
19302	2	Mastectomy, partial (eg, lumpectomy, tylectomy, quadrantectomy, segmentectomy); with axillary lymphadenectomy
19303	2	Mastectomy, simple, complete
19304	2	Mastectomy, subcutaneous
19307	3	Mastectomy, modified radical, including axillary lymph nodes, with or without pectoralis minor muscle, but excluding pectoralis major muscle
19318	2	Reduction mammaplasty
19328	1	Removal of intact mammary implant
19342	2	Delayed insertion of breast prosthesis following mastopexy, mastectomy or in reconstruction
19350	1	Nipple/areola reconstruction
19357	2	Breast reconstruction, immediate or delayed, with tissue expander, including subsequent expansion
19371	2	Periprosthetic capsulectomy, breast
19380	2	Revision of reconstructed breast
20005	2	Incision and drainage of soft tissue abscess, subfascial (ie, involves the soft tissue below the deep fascia)
20103	2	Exploration of penetrating wound (separate procedure); extremity
20680	2	Removal of implant; deep (eg, buried wire, pin, screw, metal band, nail, rod or plate)
21025	3	Excision of bone (eg, for osteomyelitis or bone abscess); mandible
21215	3	Graft, bone; mandible (includes obtaining graft)
21390	3	Open treatment of orbital floor blowout fracture; periorbital approach, with alloplastic or other implant
21462	2	Open treatment of mandibular fracture; with interdental fixation
21470	3	Open treatment of complicated mandibular fracture by multiple surgical approaches including internal fixation, interdental fixation, and/or wiring of dentures or splints
21501	2	Incision and drainage, deep abscess or hematoma, soft tissues of neck or thorax
21627	3	Sternal debridement
21750	3	Closure of median sternotomy separation with or without debridement (separate procedure)
22551	2	Arthrodesis, anterior interbody, including disc space preparation, discectomy, osteophytectomy and decompression of spinal cord and/or nerve roots; cervical below C2
22554	2	Arthrodesis, anterior interbody technique, including minimal discectomy to prepare interspace (other than for decompression); cervical below C2
22558	3	Arthrodesis, anterior interbody technique, including minimal discectomy to prepare interspace (other than for decompression); lumbar
22600	3	Arthrodesis, posterior or posterolateral technique, single level; cervical below C2 segment
22612	3	Arthrodesis, posterior or posterolateral technique, single level; lumbar (with lateral transverse technique, when performed)
22630	3	Arthrodesis, posterior interbody technique, including laminectomy and/or discectomy to prepare interspace (other than for decompression), single interspace; lumbar
22633	3	Arthrodesis, combined posterior or posterolateral technique with posterior interbody technique including laminectomy and/or discectomy sufficient to prepare interspace (other than for decompression), single interspace and segment; lumbar
22856	3	Total disc arthroplasty (artificial disc), anterior approach, including discectomy with end plate preparation (includes osteophytectomy for nerve root or spinal cord decompression and microdissection); single interspace, cervical
22899	3	Unlisted procedure, spine

© 2020 Shinall MC Jr et al. *JAMA Network Open.*

CPT Code	OSS	CPT Description
23030	2	Incision and drainage, shoulder area; deep abscess or hematoma
23120	1	Claviculectomy; partial
23130	2	Acromioplasty or acromionectomy, partial, with or without coracoacromial ligament release
23410	2	Repair of ruptured musculotendinous cuff (eg, rotator cuff) open; acute
23412	2	Repair of ruptured musculotendinous cuff (eg, rotator cuff) open; chronic
23415	2	Coracoacromial ligament release, with or without acromioplasty
23420	2	Reconstruction of complete shoulder (rotator) cuff avulsion, chronic (includes acromioplasty)
23430	2	Tenodesis of long tendon of biceps
23455	2	Capsulorrhaphy, anterior; with labral repair (eg, Bankart procedure)
23470	3	Arthroplasty, glenohumeral joint; hemiarthroplasty
23472	3	Arthroplasty, glenohumeral joint; total shoulder (glenoid and proximal humeral replacement (eg, total shoulder))
23515	2	Open treatment of clavicular fracture, includes internal fixation, when performed
23550	2	Open treatment of acromioclavicular dislocation, acute or chronic;
23615	3	Open treatment of proximal humeral (surgical or anatomical neck) fracture, includes internal fixation, when performed, includes repair of tuberosity(s), when performed;
24105	2	Excision, olecranon bursa
24341	2	Repair, tendon or muscle, upper arm or elbow, each tendon or muscle, primary or secondary (excludes rotator cuff)
24342	2	Reinsertion of ruptured biceps or triceps tendon, distal, with or without tendon graft
24358	2	Tenotomy, elbow, lateral or medial (eg, epicondylitis, tennis elbow, golfer's elbow); debridement, soft tissue and/or bone, open
24359	2	Tenotomy, elbow, lateral or medial (eg, epicondylitis, tennis elbow, golfer's elbow); debridement, soft tissue and/or bone, open with tendon repair or reattachment
24515	3	Open treatment of humeral shaft fracture with plate/screws, with or without cerclage
24685	2	Open treatment of ulnar fracture, proximal end (eg, olecranon or coronoid process[es]), includes internal fixation, when performed
25000	1	Incision, extensor tendon sheath, wrist (eg, deQuervains disease)
25111	1	Excision of ganglion, wrist (dorsal or volar); primary
25112	1	Excision of ganglion, wrist (dorsal or volar); recurrent
25210	1	Carpectomy; 1 bone
25215	2	Carpectomy; all bones of proximal row
25320	2	Capsulorrhaphy or reconstruction, wrist, open (eg, capsulodesis, ligament repair, tendon transfer or graft) (includes synovectomy, capsulotomy and open reduction) for carpal instability
25440	2	Repair of nonunion, scaphoid carpal (navicular) bone, with or without radial styloidectomy (includes obtaining graft and necessary fixation)
25445	1	Arthroplasty with prosthetic replacement; trapezium
25447	1	Arthroplasty, interposition, intercarpal or carpometacarpal joints
25606	2	Percutaneous skeletal fixation of distal radial fracture or epiphyseal separation
25607	2	Open treatment of distal radial extra-articular fracture or epiphyseal separation, with internal fixation
25608	2	Open treatment of distal radial intra-articular fracture or epiphyseal separation; with internal fixation of 2 fragments
25609	2	Open treatment of distal radial intra-articular fracture or epiphyseal separation; with internal fixation of 3 or more fragments
25628	2	Open treatment of carpal scaphoid (navicular) fracture, includes internal fixation, when performed
26121	1	Fasciectomy, palm only, with or without Z-plasty, other local tissue rearrangement, or skin grafting (includes obtaining graft)

© 2020 Shinall MC Jr et al. *JAMA Network Open.*

CPT Code	OSS	CPT Description
26123	1	Fasciectomy, partial palmar with release of single digit including proximal interphalangeal joint, with or without Z-plasty, other local tissue rearrangement, or skin grafting (includes obtaining graft);
26350	1	Repair or advancement, flexor tendon, not in zone 2 digital flexor tendon sheath (eg, no man's land); primary or secondary without free graft, each tendon
26356	1	Repair or advancement, flexor tendon, in zone 2 digital flexor tendon sheath (eg, no man's land); primary, without free graft, each tendon
26410	1	Repair, extensor tendon, hand, primary or secondary; without free graft, each tendon
26418	1	Repair, extensor tendon, finger, primary or secondary; without free graft, each tendon
26440	1	Tenolysis, flexor tendon; palm OR finger, each tendon
26530	1	Arthroplasty, metacarpophalangeal joint; each joint
26531	1	Arthroplasty, metacarpophalangeal joint; with prosthetic implant, each joint
26540	1	Repair of collateral ligament, metacarpophalangeal or interphalangeal joint
26615	1	Open treatment of metacarpal fracture, single, includes internal fixation, when performed, each bone
26727	1	Percutaneous skeletal fixation of unstable phalangeal shaft fracture, proximal or middle phalanx, finger or thumb, with manipulation, each
26735	1	Open treatment of phalangeal shaft fracture, proximal or middle phalanx, finger or thumb, includes internal fixation, when performed, each
26765	1	Open treatment of distal phalangeal fracture, finger or thumb, includes internal fixation, when performed, each
26952	1	Amputation, finger or thumb, primary or secondary, any joint or phalanx, single, including neurectomies; with local advancement flaps (V-Y, hood)
26990	3	Incision and drainage, pelvis or hip joint area; deep abscess or hematoma
27125	3	Hemiarthroplasty, hip, partial (eg, femoral stem prosthesis, bipolar arthroplasty)
27130	3	Arthroplasty, acetabular and proximal femoral prosthetic replacement (total hip arthroplasty), with or without autograft or allograft
27132	3	Conversion of previous hip surgery to total hip arthroplasty, with or without autograft or allograft
27134	3	Revision of total hip arthroplasty; both components, with or without autograft or allograft
27137	3	Revision of total hip arthroplasty; acetabular component only, with or without autograft or allograft
27138	3	Revision of total hip arthroplasty; femoral component only, with or without allograft
27235	2	Percutaneous skeletal fixation of femoral fracture, proximal end, neck
27236	3	Open treatment of femoral fracture, proximal end, neck, internal fixation or prosthetic replacement
27244	3	Treatment of intertrochanteric, peritrochanteric, or subtrochanteric femoral fracture; with plate/screw type implant, with or without cerclage
27245	3	Treatment of intertrochanteric, peritrochanteric, or subtrochanteric femoral fracture; with intramedullary implant, with or without interlocking screws and/or cerclage
27299	3	Unlisted procedure, pelvis or hip joint
27301	2	Incision and drainage, deep abscess, bursa, or hematoma, thigh or knee region
27310	2	Arthrotomy, knee, with exploration, drainage, or removal of foreign body (eg, infection)
27327	2	Excision, tumor, soft tissue of thigh or knee area, subcutaneous; less than 3 cm
27331	2	Arthrotomy, knee; including joint exploration, biopsy, or removal of loose or foreign bodies
27332	2	Arthrotomy, with excision of semilunar cartilage (meniscectomy) knee; medial OR lateral
27334	2	Arthrotomy, with synovectomy, knee; anterior OR posterior
27340	2	Excision, prepatellar bursa
27380	2	Suture of infrapatellar tendon; primary
27385	2	Suture of quadriceps or hamstring muscle rupture; primary

© 2020 Shinall MC Jr et al. *JAMA Network Open.*

CPT Code	OSS	CPT Description
27427	2	Ligamentous reconstruction (augmentation), knee; extra-articular
27428	2	Ligamentous reconstruction (augmentation), knee; intra-articular (open)
27430	2	Quadricepsplasty (eg, Bennett or Thompson type)
27438	2	Arthroplasty, patella; with prosthesis
27446	2	Arthroplasty, knee, condyle and plateau; medial OR lateral compartment
27447	2	Arthroplasty, knee, condyle and plateau; medial AND lateral compartments with or without patella resurfacing (total knee arthroplasty)
27486	3	Revision of total knee arthroplasty, with or without allograft; 1 component
27487	3	Revision of total knee arthroplasty, with or without allograft; femoral and entire tibial component
27488	3	Removal of prosthesis, including total knee prosthesis, methylmethacrylate with or without insertion of spacer, knee
27506	3	Open treatment of femoral shaft fracture, with or without external fixation, with insertion of intramedullary implant, with or without cerclage and/or locking screws
27514	3	Open treatment of femoral fracture, distal end, medial or lateral condyle, includes internal fixation, when performed
27524	2	Open treatment of patellar fracture, with internal fixation and/or partial or complete patellectomy and soft tissue repair
27535	3	Open treatment of tibial fracture, proximal (plateau); unicondylar, includes internal fixation, when performed
27590	3	Amputation, thigh, through femur, any level;
27592	3	Amputation, thigh, through femur, any level; open, circular (guillotine)
27594	3	Amputation, thigh, through femur, any level; secondary closure or scar revision
27596	3	Amputation, thigh, through femur, any level; re-amputation
27598	3	Disarticulation at knee
27599	2	Unlisted procedure, femur or knee
27602	2	Decompression fasciotomy, leg; anterior and/or lateral, and posterior compartment(s)
27603	2	Incision and drainage, leg or ankle; deep abscess or hematoma
27610	2	Arthrotomy, ankle, including exploration, drainage, or removal of foreign body
27620	2	Arthrotomy, ankle, with joint exploration, with or without biopsy, with or without removal of loose or foreign body
27630	2	Excision of lesion of tendon sheath or capsule (eg, cyst or ganglion), leg and/or ankle
27635	2	Excision or curettage of bone cyst or benign tumor, tibia or fibula;
27650	2	Repair, primary, open or percutaneous, ruptured Achilles tendon;
27652	2	Repair, primary, open or percutaneous, ruptured Achilles tendon; with graft (includes obtaining graft)
27654	2	Repair, secondary, Achilles tendon, with or without graft
27675	2	Repair, dislocating peroneal tendons; without fibular osteotomy
27685	2	Lengthening or shortening of tendon, leg or ankle; single tendon (separate procedure)
27687	1	Gastrocnemius recession (eg, Strayer procedure)
27695	1	Repair, primary, disrupted ligament, ankle; collateral
27698	1	Repair, secondary, disrupted ligament, ankle, collateral (eg, Watson-Jones procedure)
27702	2	Arthroplasty, ankle; with implant (total ankle)
27704	2	Removal of ankle implant
27759	2	Treatment of tibial shaft fracture (with or without fibular fracture) by intramedullary implant, with or without interlocking screws and/or cerclage
27766	2	Open treatment of medial malleolus fracture, includes internal fixation, when performed
27792	2	Open treatment of distal fibular fracture (lateral malleolus), includes internal fixation, when performed

© 2020 Shinall MC Jr et al. *JAMA Network Open.*

CPT Code	OSS	CPT Description
27814	2	Open treatment of bimalleolar ankle fracture (eg, lateral and medial malleoli, or lateral and posterior malleoli, or medial and posterior malleoli), includes internal fixation, when performed
27822	2	Open treatment of trimalleolar ankle fracture, includes internal fixation, when performed, medial and/or lateral malleolus; without fixation of posterior lip
27827	2	Open treatment of fracture of weight bearing articular surface/portion of distal tibia (eg, pilon or tibial plafond), with internal fixation, when performed; of tibia only
27829	2	Open treatment of distal tibiofibular joint (syndesmosis) disruption, includes internal fixation, when performed
27870	2	Arthrodesis, ankle, open
27880	3	Amputation, leg, through tibia and fibula
27881	3	Amputation, leg, through tibia and fibula; with immediate fitting technique including application of first cast
27882	3	Amputation, leg, through tibia and fibula; open, circular (guillotine)
27884	2	Amputation, leg, through tibia and fibula; secondary closure or scar revision
27886	3	Amputation, leg, through tibia and fibula; re-amputation
27888	2	Amputation, ankle, through malleoli of tibia and fibula (eg, Syme, Pirogoff type procedures), with plastic closure and resection of nerves
27889	2	Ankle disarticulation
28002	2	Incision and drainage below fascia, with or without tendon sheath involvement, foot; single bursal space
28035	1	Release, tarsal tunnel (posterior tibial nerve decompression)
28800	2	Amputation, foot; midtarsal (eg, Chopart type procedure)
29806	2	Arthroscopy, shoulder, surgical; capsulorrhaphy
29807	2	Arthroscopy, shoulder, surgical; repair of SLAP lesion
29822	2	Arthroscopy, shoulder, surgical; debridement, limited
29823	2	Arthroscopy, shoulder, surgical; debridement, extensive
29824	2	Arthroscopy, shoulder, surgical; distal claviculectomy including distal articular surface (Mumford procedure)
29825	2	Arthroscopy, shoulder, surgical; with lysis and resection of adhesions, with or without manipulation
29826	2	Arthroscopy, shoulder, surgical; decompression of subacromial space with partial acromioplasty, with coracoacromial ligament (ie, arch) release, when performed (List separately in addition to code for primary procedure)
29827	2	Arthroscopy, shoulder, surgical; with rotator cuff repair
29828	2	Arthroscopy, shoulder, surgical; biceps tenodesis
29846	1	Arthroscopy, wrist, surgical; excision and/or repair of triangular fibrocartilage and/or joint debridement
29870	2	Arthroscopy, knee, diagnostic, with or without synovial biopsy (separate procedure)
29871	2	Arthroscopy, knee, surgical; for infection, lavage and drainage
29873	2	Arthroscopy, knee, surgical; with lateral release
29874	2	Arthroscopy, knee, surgical; for removal of loose body or foreign body (eg, osteochondritis dissecans fragmentation, chondral fragmentation)
29875	2	Arthroscopy, knee, surgical; synovectomy, limited (eg, plica or shelf resection) (separate procedure)
29876	2	Arthroscopy, knee, surgical; synovectomy, major, 2 or more compartments (eg, medial or lateral)
29877	2	Arthroscopy, knee, surgical; debridement/shaving of articular cartilage (chondroplasty)
29879	2	Arthroscopy, knee, surgical; abrasion arthroplasty (includes chondroplasty where necessary) or multiple drilling or microfracture

© 2020 Shinall MC Jr et al. *JAMA Network Open.*

CPT Code	OSS	CPT Description
29880	2	Arthroscopy, knee, surgical; with meniscectomy (medial AND lateral, including any meniscal shaving) including debridement/shaving of articular cartilage (chondroplasty), same or separate compartment(s), when performed
29881	2	Arthroscopy, knee, surgical; with meniscectomy (medial OR lateral, including any meniscal shaving) including debridement/shaving of articular cartilage (chondroplasty), same or separate compartment(s), when performed
29882	2	Arthroscopy, knee, surgical; with meniscus repair (medial OR lateral)
29883	2	Arthroscopy, knee, surgical; with meniscus repair (medial AND lateral)
29884	2	Arthroscopy, knee, surgical; with lysis of adhesions, with or without manipulation (separate procedure)
29888	2	Arthroscopically aided anterior cruciate ligament repair/augmentation or reconstruction
29891	2	Arthroscopy, ankle, surgical, excision of osteochondral defect of talus and/or tibia, including drilling of the defect
29893	1	Endoscopic plantar fasciotomy
29895	1	Arthroscopy, ankle (tibiotalar and fibulotalar joints), surgical; synovectomy, partial
29897	1	Arthroscopy, ankle (tibiotalar and fibulotalar joints), surgical; debridement, limited
29898	1	Arthroscopy, ankle (tibiotalar and fibulotalar joints), surgical; debridement, extensive
29999	2	Unlisted procedure, arthroscopy
30420	2	Rhinoplasty, primary; including major septal repair
30520	2	Septoplasty or submucous resection, with or without cartilage scoring, contouring or replacement with graft
31276	2	Nasal/sinus endoscopy, surgical with frontal sinus exploration, with or without removal of tissue from frontal sinus
31360	4	Laryngectomy; total, without radical neck dissection
31365	4	Laryngectomy; total, with radical neck dissection
31599	2	Unlisted procedure, larynx
31611	1	Construction of tracheoesophageal fistula and subsequent insertion of an alaryngeal speech prosthesis (eg, voice button, Blom-Singer prosthesis)
31622	1	Bronchoscopy, rigid or flexible, including fluoroscopic guidance, when performed; diagnostic, with cell washing, when performed (separate procedure)
31820	1	Surgical closure tracheostomy or fistula; without plastic repair
32100	4	Thoracotomy; with exploration
32120	5	Thoracotomy; for postoperative complications
32220	4	Decortication, pulmonary (separate procedure); total
32225	3	Decortication, pulmonary (separate procedure); partial
32320	4	Decortication and parietal pleurectomy
32440	5	Removal of lung, pneumonectomy;
32480	4	Removal of lung, other than pneumonectomy; single lobe (lobectomy)
32482	4	Removal of lung, other than pneumonectomy; 2 lobes (bilobectomy)
32484	4	Removal of lung, other than pneumonectomy; single segment (segmentectomy)
32505	4	Thoracotomy; with therapeutic wedge resection (eg, mass, nodule), initial
32650	3	Thoracoscopy, surgical; with pleurodesis (eg, mechanical or chemical)
32651	4	Thoracoscopy, surgical; with partial pulmonary decortication
32652	4	Thoracoscopy, surgical; with total pulmonary decortication, including intrapleural pneumonolysis
32655	4	Thoracoscopy, surgical; with resection-plication of bullae, includes any pleural procedure when performed
32663	4	Thoracoscopy, surgical; with lobectomy (single lobe)
32666	3	Thoracoscopy, surgical; with therapeutic wedge resection (eg, mass, nodule), initial unilateral

© 2020 Shinall MC Jr et al. *JAMA Network Open.*

CPT Code	OSS	CPT Description
32669	3	Thoracoscopy, surgical; with removal of a single lung segment (segmentectomy)
33025	3	Creation of pericardial window or partial resection for drainage
34201	2	Embolectomy or thrombectomy, with or without catheter; femoropopliteal, aortoiliac artery, by leg incision
34800	2	Endovascular repair of infrarenal abdominal aortic aneurysm or dissection; using aorto-aortic tube prosthesis
34802	3	Endovascular repair of infrarenal abdominal aortic aneurysm or dissection; using modular bifurcated prosthesis (1 docking limb)
34803	3	Endovascular repair of infrarenal abdominal aortic aneurysm or dissection; using modular bifurcated prosthesis (2 docking limbs)
34804	3	Endovascular repair of infrarenal abdominal aortic aneurysm or dissection; using unibody bifurcated prosthesis
34805	3	Endovascular repair of infrarenal abdominal aortic aneurysm or dissection; using aorto-uniiliac or aorto-unifemoral prosthesis
34812	2	Open femoral artery exposure for delivery of endovascular prosthesis, by groin incision, unilateral
34825	2	Placement of proximal or distal extension prosthesis for endovascular repair of infrarenal abdominal aortic or iliac aneurysm, false aneurysm, or dissection; initial vessel
34900	2	Endovascular repair of iliac artery (eg, aneurysm, pseudoaneurysm, arteriovenous malformation, trauma) using ilio-iliac tube endoprosthesis
35081	5	Direct repair of aneurysm, pseudoaneurysm, or excision (partial or total) and graft insertion, with or without patch graft; for aneurysm, pseudoaneurysm, and associated occlusive disease, abdominal aorta
35091	5	Direct repair of aneurysm, pseudoaneurysm, or excision (partial or total) and graft insertion, with or without patch graft; for aneurysm, pseudoaneurysm, and associated occlusive disease, abdominal aorta involving visceral vessels (mesenteric, celiac, renal)
35102	5	Direct repair of aneurysm, pseudoaneurysm, or excision (partial or total) and graft insertion, with or without patch graft; for aneurysm, pseudoaneurysm, and associated occlusive disease, abdominal aorta involving iliac vessels (common, hypogastric, exter
35141	3	Direct repair of aneurysm, pseudoaneurysm, or excision (partial or total) and graft insertion, with or without patch graft; for aneurysm, pseudoaneurysm, and associated occlusive disease, common femoral artery (profunda femoris, superficial femoral)
35151	3	Direct repair of aneurysm, pseudoaneurysm, or excision (partial or total) and graft insertion, with or without patch graft; for aneurysm, pseudoaneurysm, and associated occlusive disease, popliteal artery
35226	2	Repair blood vessel, direct; lower extremity
35301	3	Thromboendarterectomy, including patch graft, if performed; carotid, vertebral, subclavian, by neck incision
35302	2	Thromboendarterectomy, including patch graft, if performed; superficial femoral artery
35371	2	Thromboendarterectomy, including patch graft, if performed; common femoral
35372	2	Thromboendarterectomy, including patch graft, if performed; deep (profunda) femoral
35540	4	Bypass graft, with vein; aortobifemoral
35556	3	Bypass graft, with vein; femoral-popliteal
35558	3	Bypass graft, with vein; femoral-femoral
35566	3	Bypass graft, with vein; femoral-anterior tibial, posterior tibial, peroneal artery or other distal vessels
35571	3	Bypass graft, with vein; popliteal-tibial, -peroneal artery or other distal vessels
35583	3	In-situ vein bypass; femoral-popliteal
35585	3	In-situ vein bypass; femoral-anterior tibial, posterior tibial, or peroneal artery
35606	3	Bypass graft, with other than vein; carotid-subclavian
35621	3	Bypass graft, with other than vein; axillary-femoral

© 2020 Shinall MC Jr et al. *JAMA Network Open.*

CPT Code	OSS	CPT Description
35646	4	Bypass graft, with other than vein; aortobifemoral
35654	3	Bypass graft, with other than vein; axillary-femoral-femoral
35656	3	Bypass graft, with other than vein; femoral-popliteal
35661	3	Bypass graft, with other than vein; femoral-femoral
35665	3	Bypass graft, with other than vein; iliofemoral
35666	3	Bypass graft, with other than vein; femoral-anterior tibial, posterior tibial, or peroneal artery
35800	3	Exploration for postoperative hemorrhage, thrombosis or infection; neck
35820	4	Exploration for postoperative hemorrhage, thrombosis or infection; chest
35840	4	Exploration for postoperative hemorrhage, thrombosis or infection; abdomen
35860	3	Exploration for postoperative hemorrhage, thrombosis or infection; extremity
35875	1	Thrombectomy of arterial or venous graft (other than hemodialysis graft or fistula);
35876	2	Thrombectomy of arterial or venous graft (other than hemodialysis graft or fistula); with revision of arterial or venous graft
35879	2	Revision, lower extremity arterial bypass, without thrombectomy, open; with vein patch angioplasty
35883	2	Revision, femoral anastomosis of synthetic arterial bypass graft in groin, open; with nonautogenous patch graft (eg, Dacron, ePTFE, bovine pericardium)
35903	2	Excision of infected graft; extremity
36246	1	Selective catheter placement, arterial system; initial second order abdominal, pelvic, or lower extremity artery branch, within a vascular family
36247	1	Selective catheter placement, arterial system; initial third order or more selective abdominal, pelvic, or lower extremity artery branch, within a vascular family
36475	1	Endovenous ablation therapy of incompetent vein, extremity, inclusive of all imaging guidance and monitoring, percutaneous, radiofrequency; first vein treated
36478	1	Endovenous ablation therapy of incompetent vein, extremity, inclusive of all imaging guidance and monitoring, percutaneous, laser; first vein treated
36561	1	Insertion of tunneled centrally inserted central venous access device, with subcutaneous port; age 5 years or older
37215	2	Transcatheter placement of intravascular stent(s), cervical carotid artery, open or percutaneous, including angioplasty, when performed, and radiological supervision and interpretation; with distal embolic protection
37220	2	Revascularization, endovascular, open or percutaneous, iliac artery, unilateral, initial vessel; with transluminal angioplasty
37221	2	Revascularization, endovascular, open or percutaneous, iliac artery, unilateral, initial vessel; with transluminal stent placement(s), includes angioplasty within the same vessel, when performed
37224	2	Revascularization, endovascular, open or percutaneous, femoral, popliteal artery(s), unilateral; with transluminal angioplasty
37226	2	Revascularization, endovascular, open or percutaneous, femoral, popliteal artery(s), unilateral; with transluminal stent placement(s), includes angioplasty within the same vessel, when performed
37228	2	Revascularization, endovascular, open or percutaneous, tibial, peroneal artery, unilateral, initial vessel; with transluminal angioplasty
37722	1	Ligation, division, and stripping, long (greater) saphenous veins from saphenofemoral junction to knee or below
37765	1	Stab phlebectomy of varicose veins, 1 extremity; 10-20 stab incisions
37766	2	Stab phlebectomy of varicose veins, 1 extremity; more than 20 incisions
37799	2	Unlisted procedure, vascular surgery
38100	3	Splenectomy; total (separate procedure)
38120	3	Laparoscopy, surgical, splenectomy

© 2020 Shinall MC Jr et al. *JAMA Network Open.*

CPT Code	OSS	CPT Description
38720	2	Cervical lymphadenectomy (complete)
38724	3	Cervical lymphadenectomy (modified radical neck dissection)
38740	2	Axillary lymphadenectomy; superficial
38745	3	Axillary lymphadenectomy; complete
38760	2	Inguinofemoral lymphadenectomy, superficial, including cloquets node (separate procedure)
39010	3	Mediastinotomy with exploration, drainage, removal of foreign body, or biopsy; transthoracic approach, including either transthoracic or median sternotomy
39400	2	Mediastinoscopy, includes biopsy(ies), when performed
40510	1	Excision of lip; transverse wedge excision with primary closure
40812	1	Excision of lesion of mucosa and submucosa, vestibule of mouth; with simple repair
41110	1	Excision of lesion of tongue without closure
41112	1	Excision of lesion of tongue with closure; anterior two-thirds
41116	2	Excision, lesion of floor of mouth
41120	2	Glossectomy; less than one-half tongue
41135	3	Glossectomy; partial, with unilateral radical neck dissection
41155	4	Glossectomy; composite procedure with resection floor of mouth, mandibular resection, and radical neck dissection (Commando type)
42104	2	Excision, lesion of palate, uvula; without closure
42140	2	Uvulectomy, excision of uvula
42145	2	Palatopharyngoplasty (eg, uvulopalatopharyngoplasty, uvulopharyngoplasty)
42410	2	Excision of parotid tumor or parotid gland; lateral lobe, without nerve dissection
42415	2	Excision of parotid tumor or parotid gland; lateral lobe, with dissection and preservation of facial nerve
42420	2	Excision of parotid tumor or parotid gland; total, with dissection and preservation of facial nerve
42426	3	Excision of parotid tumor or parotid gland; total, with unilateral radical neck dissection
42440	2	Excision of submandibular (submaxillary) gland
42808	2	Excision or destruction of lesion of pharynx, any method
42826	2	Tonsillectomy, primary or secondary; age 12 or over
43107	5	Total or near total esophagectomy, without thoracotomy; with pharyngogastrostomy or cervical esophagogastrostomy, with or without pyloroplasty (transhiatal)
43112	5	Total or near total esophagectomy, with thoracotomy; with pharyngogastrostomy or cervical esophagogastrostomy, with or without pyloroplasty
43117	5	Partial esophagectomy, distal two-thirds, with thoracotomy and separate abdominal incision, with or without proximal gastrectomy; with thoracic esophagogastrostomy, with or without pyloroplasty (Ivor Lewis)
43130	3	Diverticulectomy of hypopharynx or esophagus, with or without myotomy; cervical approach
43279	2	Laparoscopy, surgical, esophagomyotomy (Heller type), with fundoplasty, when performed
43280	3	Laparoscopy, surgical, esophagogastric fundoplasty (eg, Nissen, Toupet procedures)
43281	3	Laparoscopy, surgical, repair of paraesophageal hernia, includes fundoplasty, when performed; without implantation of mesh
43282	3	Laparoscopy, surgical, repair of paraesophageal hernia, includes fundoplasty, when performed; with implantation of mesh
43610	3	Excision, local; ulcer or benign tumor of stomach
43631	4	Gastrectomy, partial, distal; with gastroduodenostomy
43632	4	Gastrectomy, partial, distal; with gastrojejunostomy
43633	4	Gastrectomy, partial, distal; with Roux-en-Y reconstruction
43644	3	Laparoscopy, surgical, gastric restrictive procedure; with gastric bypass and Roux-en-Y gastroenterostomy (roux limb 150 cm or less)

© 2020 Shinall MC Jr et al. *JAMA Network Open.*

CPT Code	OSS	CPT Description
43659	3	Unlisted laparoscopy procedure, stomach
43775	3	Laparoscopy, surgical, gastric restrictive procedure; longitudinal gastrectomy (ie, sleeve gastrectomy)
43820	3	Gastrojejunostomy; without vagotomy
43840	3	Gastrorrhaphy, suture of perforated duodenal or gastric ulcer, wound, or injury
44005	3	Enterolysis (freeing of intestinal adhesion) (separate procedure)
44050	3	Reduction of volvulus, intussusception, internal hernia, by laparotomy
44120	3	Enterectomy, resection of small intestine; single resection and anastomosis
44125	3	Enterectomy, resection of small intestine; with enterostomy
44130	3	Enteroenterostomy, anastomosis of intestine, with or without cutaneous enterostomy (separate procedure)
44140	4	Colectomy, partial; with anastomosis
44141	4	Colectomy, partial; with skin level cecostomy or colostomy
44143	4	Colectomy, partial; with end colostomy and closure of distal segment (Hartmann type procedure)
44144	4	Colectomy, partial; with resection, with colostomy or ileostomy and creation of mucofistula
44145	4	Colectomy, partial; with coloproctostomy (low pelvic anastomosis)
44146	4	Colectomy, partial; with coloproctostomy (low pelvic anastomosis), with colostomy
44150	4	Colectomy, total, abdominal, without proctectomy; with ileostomy or ileoproctostomy
44155	4	Colectomy, total, abdominal, with proctectomy; with ileostomy
44160	4	Colectomy, partial, with removal of terminal ileum with ileocolostomy
44180	3	Laparoscopy, surgical, enterolysis (freeing of intestinal adhesion) (separate procedure)
44188	3	Laparoscopy, surgical, colostomy or skin level cecostomy
44202	3	Laparoscopy, surgical; enterectomy, resection of small intestine, single resection and anastomosis
44204	3	Laparoscopy, surgical; colectomy, partial, with anastomosis
44205	3	Laparoscopy, surgical; colectomy, partial, with removal of terminal ileum with ileocolostomy
44206	3	Laparoscopy, surgical; colectomy, partial, with end colostomy and closure of distal segment (Hartmann type procedure)
44207	3	Laparoscopy, surgical; colectomy, partial, with anastomosis, with coloproctostomy (low pelvic anastomosis)
44227	3	Laparoscopy, surgical, closure of enterostomy, large or small intestine, with resection and anastomosis
44310	3	Ileostomy or jejunostomy, non-tube
44312	2	Revision of ileostomy; simple (release of superficial scar) (separate procedure)
44314	3	Revision of ileostomy; complicated (reconstruction in-depth) (separate procedure)
44320	3	Colostomy or skin level cecostomy;
44340	2	Revision of colostomy; simple (release of superficial scar) (separate procedure)
44345	3	Revision of colostomy; complicated (reconstruction in-depth) (separate procedure)
44346	3	Revision of colostomy; with repair of paracolostomy hernia (separate procedure)
44602	3	Suture of small intestine (enterorrhaphy) for perforated ulcer, diverticulum, wound, injury or rupture; single perforation
44604	3	Suture of large intestine (colorrhaphy) for perforated ulcer, diverticulum, wound, injury or rupture (single or multiple perforations); without colostomy
44620	3	Closure of enterostomy, large or small intestine;
44625	3	Closure of enterostomy, large or small intestine; with resection and anastomosis other than colorectal
44626	3	Closure of enterostomy, large or small intestine; with resection and colorectal anastomosis (eg, closure of Hartmann type procedure)

CPT Code	OSS	CPT Description
44640	4	Closure of intestinal cutaneous fistula
44661	4	Closure of enterovesical fistula; with intestine and/or bladder resection
44950	2	Appendectomy;
44960	3	Appendectomy; for ruptured appendix with abscess or generalized peritonitis
44970	2	Laparoscopy, surgical, appendectomy
45005	2	Incision and drainage of submucosal abscess, rectum
45110	4	Proctectomy; complete, combined abdominoperineal, with colostomy
45111	4	Proctectomy; partial resection of rectum, transabdominal approach
45395	4	Laparoscopy, surgical; proctectomy, complete, combined abdominoperineal, with colostomy
46020	1	Placement of seton
46040	1	Incision and drainage of ischiorectal and/or perirectal abscess (separate procedure)
46045	1	Incision and drainage of intramural, intramuscular, or submucosal abscess, transanal, under anesthesia
46060	1	Incision and drainage of ischiorectal or intramural abscess, with fistulectomy or fistulotomy, submuscular, with or without placement of seton
46270	1	Surgical treatment of anal fistula (fistulectomy/fistulotomy); subcutaneous
46947	1	Hemorrhoidopexy (eg, for prolapsing internal hemorrhoids) by stapling
46999	1	Unlisted procedure, anus
47120	4	Hepatectomy, resection of liver; partial lobectomy
47135	5	Liver allotransplantation; orthotopic, partial or whole, from cadaver or living donor, any age
47370	3	Laparoscopy, surgical, ablation of 1 or more liver tumor(s); radiofrequency
47379	3	Unlisted laparoscopic procedure, liver
47562	3	Laparoscopy, surgical; cholecystectomy
47563	3	Laparoscopy, surgical; cholecystectomy with cholangiography
47564	3	Laparoscopy, surgical; cholecystectomy with exploration of common duct
47600	3	Cholecystectomy;
47605	3	Cholecystectomy; with cholangiography
47610	3	Cholecystectomy with exploration of common duct;
48140	4	Pancreatectomy, distal subtotal, with or without splenectomy; without pancreaticojejunostomy
48150	5	Pancreatectomy, proximal subtotal with total duodenectomy, partial gastrectomy, choledochoenterostomy and gastrojejunostomy (Whipple-type procedure); with pancreatojejunostomy
48153	5	Pancreatectomy, proximal subtotal with near-total duodenectomy, choledochoenterostomy and duodenojejunostomy (pylorus-sparing, Whipple-type procedure); with pancreatojejunostomy
49000	3	Exploratory laparotomy, exploratory celiotomy with or without biopsy(s) (separate procedure)
49002	3	Reopening of recent laparotomy
49010	3	Exploration, retroperitoneal area with or without biopsy(s) (separate procedure)
49020	3	Drainage of peritoneal abscess or localized peritonitis, exclusive of appendiceal abscess; open
49203	3	Excision or destruction, open, intra-abdominal tumors, cysts or endometriomas, 1 or more peritoneal, mesenteric, or retroperitoneal primary or secondary tumors; largest tumor 5 cm diameter or less
49321	2	Laparoscopy, surgical; with biopsy (single or multiple)
49324	2	Laparoscopy, surgical; with insertion of tunneled intraperitoneal catheter
49329	2	Unlisted laparoscopy procedure, abdomen, peritoneum and omentum
49402	2	Removal of peritoneal foreign body from peritoneal cavity
49505	2	Repair initial inguinal hernia, age 5 years or older; reducible
49507	2	Repair initial inguinal hernia, age 5 years or older; incarcerated or strangulated

© 2020 Shinall MC Jr et al. *JAMA Network Open.*

CPT Code	OSS	CPT Description
49520	2	Repair recurrent inguinal hernia, any age; reducible
49521	2	Repair recurrent inguinal hernia, any age; incarcerated or strangulated
49525	2	Repair inguinal hernia, sliding, any age
49550	2	Repair initial femoral hernia, any age; reducible
49553	3	Repair initial femoral hernia, any age; incarcerated or strangulated
49560	2	Repair initial incisional or ventral hernia; reducible
49561	3	Repair initial incisional or ventral hernia; incarcerated or strangulated
49565	2	Repair recurrent incisional or ventral hernia; reducible
49566	3	Repair recurrent incisional or ventral hernia; incarcerated or strangulated
49570	2	Repair epigastric hernia (eg, preperitoneal fat); reducible (separate procedure)
49572	2	Repair epigastric hernia (eg, preperitoneal fat); incarcerated or strangulated
49585	2	Repair umbilical hernia, age 5 years or older; reducible
49587	3	Repair umbilical hernia, age 5 years or older; incarcerated or strangulated
49590	2	Repair spigelian hernia
49650	2	Laparoscopy, surgical; repair initial inguinal hernia
49651	2	Laparoscopy, surgical; repair recurrent inguinal hernia
49652	2	Laparoscopy, surgical, repair, ventral, umbilical, spigelian or epigastric hernia (includes mesh insertion, when performed); reducible
49653	2	Laparoscopy, surgical, repair, ventral, umbilical, spigelian or epigastric hernia (includes mesh insertion, when performed); incarcerated or strangulated
49654	2	Laparoscopy, surgical, repair, incisional hernia (includes mesh insertion, when performed); reducible
49655	3	Laparoscopy, surgical, repair, incisional hernia (includes mesh insertion, when performed); incarcerated or strangulated
49656	2	Laparoscopy, surgical, repair, recurrent incisional hernia (includes mesh insertion, when performed); reducible
49659	2	Unlisted laparoscopy procedure, hernioplasty, herniorrhaphy, herniotomy
49900	3	Suture, secondary, of abdominal wall for evisceration or dehiscence
49999	3	Unlisted procedure, abdomen, peritoneum and omentum
50220	3	Nephrectomy, including partial ureterectomy, any open approach including rib resection;
50230	4	Nephrectomy, including partial ureterectomy, any open approach including rib resection; radical, with regional lymphadenectomy and/or vena caval thrombectomy
50234	4	Nephrectomy with total ureterectomy and bladder cuff; through same incision
50240	4	Nephrectomy, partial
50360	3	Renal allotransplantation, implantation of graft; without recipient nephrectomy
50543	2	Laparoscopy, surgical; partial nephrectomy
50545	3	Laparoscopy, surgical; radical nephrectomy (includes removal of Gerota's fascia and surrounding fatty tissue, removal of regional lymph nodes, and adrenalectomy)
50546	3	Laparoscopy, surgical; nephrectomy, including partial ureterectomy
50548	3	Laparoscopy, surgical; nephrectomy with total ureterectomy
51040	2	Cystostomy, cystotomy with drainage
51050	2	Cystolithotomy, cystotomy with removal of calculus, without vesical neck resection
51590	4	Cystectomy, complete, with ureteroileal conduit or sigmoid bladder, including intestine anastomosis;
51595	4	Cystectomy, complete, with ureteroileal conduit or sigmoid bladder, including intestine anastomosis; with bilateral pelvic lymphadenectomy, including external iliac, hypogastric, and obturator nodes

© 2020 Shinall MC Jr et al. *JAMA Network Open.*

CPT Code	OSS	CPT Description
51596	4	Cystectomy, complete, with continent diversion, any open technique, using any segment of small and/or large intestine to construct neobladder
52000	1	Cystourethroscopy (separate procedure)
52005	1	Cystourethroscopy, with ureteral catheterization, with or without irrigation, instillation, or ureteropyelography, exclusive of radiologic service;
52204	1	Cystourethroscopy, with biopsy(s)
52224	1	Cystourethroscopy, with fulguration (including cryosurgery or laser surgery) or treatment of MINOR (less than 0.5 cm) lesion(s) with or without biopsy
52234	1	Cystourethroscopy, with fulguration (including cryosurgery or laser surgery) and/or resection of; SMALL bladder tumor(s) (0.5 up to 2.0 cm)
52235	1	Cystourethroscopy, with fulguration (including cryosurgery or laser surgery) and/or resection of; MEDIUM bladder tumor(s) (2.0 to 5.0 cm)
52240	2	Cystourethroscopy, with fulguration (including cryosurgery or laser surgery) and/or resection of; LARGE bladder tumor(s)
52317	1	Litholapaxy: crushing or fragmentation of calculus by any means in bladder and removal of fragments; simple or small (less than 2.5 cm)
52332	1	Cystourethroscopy, with insertion of indwelling ureteral stent (eg, Gibbons or double-J type)
52341	1	Cystourethroscopy; with treatment of ureteral stricture (eg, balloon dilation, laser, electrocautery, and incision)
52344	1	Cystourethroscopy with ureteroscopy; with treatment of ureteral stricture (eg, balloon dilation, laser, electrocautery, and incision)
52351	1	Cystourethroscopy, with ureteroscopy and/or pyeloscopy; diagnostic
52353	1	Cystourethroscopy, with ureteroscopy and/or pyeloscopy; with lithotripsy (ureteral catheterization is included)
52450	2	Transurethral incision of prostate
52500	1	Transurethral resection of bladder neck (separate procedure)
52601	2	Transurethral electrosurgical resection of prostate, including control of postoperative bleeding, complete (vasectomy, meatotomy, cystourethroscopy, urethral calibration and/or dilation, and internal urethrotomy are included)
52630	2	Transurethral resection; residual or regrowth of obstructive prostate tissue including control of postoperative bleeding, complete (vasectomy, meatotomy, cystourethroscopy, urethral calibration and/or dilation, and internal urethrotomy are included)
52640	1	Transurethral resection; of postoperative bladder neck contracture
52648	2	Laser vaporization of prostate, including control of postoperative bleeding, complete (vasectomy, meatotomy, cystourethroscopy, urethral calibration and/or dilation, internal urethrotomy and transurethral resection of prostate are included if performed)
52649	2	Laser enucleation of the prostate with morcellation, including control of postoperative bleeding, complete (vasectomy, meatotomy, cystourethroscopy, urethral calibration and/or dilation, internal urethrotomy and transurethral resection of prostate are inc
53410	2	Urethroplasty, 1-stage reconstruction of male anterior urethra
53440	2	Sling operation for correction of male urinary incontinence (eg, fascia or synthetic)
53445	2	Insertion of inflatable urethral/bladder neck sphincter, including placement of pump, reservoir, and cuff
53446	2	Removal of inflatable urethral/bladder neck sphincter, including pump, reservoir, and cuff
53447	2	Removal and replacement of inflatable urethral/bladder neck sphincter including pump, reservoir, and cuff at the same operative session
53899	1	Unlisted procedure, urinary system
54065	1	Destruction of lesion(s), penis (eg, condyloma, papilloma, molluscum contagiosum, herpetic vesicle), extensive (eg, laser surgery, electrosurgery, cryosurgery, chemosurgery)
54120	2	Amputation of penis; partial
54360	2	Plastic operation on penis to correct angulation

© 2020 Shinall MC Jr et al. *JAMA Network Open.*

CPT Code	OSS	CPT Description
54405	2	Insertion of multi-component, inflatable penile prosthesis, including placement of pump, cylinders, and reservoir
54840	1	Excision of spermatocele, with or without epididymectomy
54860	1	Epididymectomy; unilateral
55040	1	Excision of hydrocele; unilateral
55041	1	Excision of hydrocele; bilateral
55060	1	Repair of tunica vaginalis hydrocele (Bottle type)
55100	1	Drainage of scrotal wall abscess
55110	1	Scrotal exploration
55530	1	Excision of varicocele or ligation of spermatic veins for varicocele; (separate procedure)
55810	4	Prostatectomy, perineal radical;
55821	3	Prostatectomy (including control of postoperative bleeding, vasectomy, meatotomy, urethral calibration and/or dilation, and internal urethrotomy); suprapubic, subtotal, 1 or 2 stages
55831	3	Prostatectomy (including control of postoperative bleeding, vasectomy, meatotomy, urethral calibration and/or dilation, and internal urethrotomy); retropubic, subtotal
55840	3	Prostatectomy, retropubic radical, with or without nerve sparing;
55842	4	Prostatectomy, retropubic radical, with or without nerve sparing; with lymph node biopsy(s) (limited pelvic lymphadenectomy)
55845	4	Prostatectomy, retropubic radical, with or without nerve sparing; with bilateral pelvic lymphadenectomy, including external iliac, hypogastric, and obturator nodes
55866	3	Laparoscopy, surgical prostatectomy, retropubic radical, including nerve sparing, includes robotic assistance, when performed
55873	2	Cryosurgical ablation of the prostate (includes ultrasonic guidance and monitoring)
55876	1	Placement of interstitial device(s) for radiation therapy guidance (eg, fiducial markers, dosimeter), prostate (via needle, any approach), single or multiple
57288	2	Sling operation for stress incontinence (eg, fascia or synthetic)
57460	1	Colposcopy of the cervix including upper/adjacent vagina; with loop electrode biopsy(s) of the cervix
57522	1	Conization of cervix, with or without fulguration, with or without dilation and curettage, with or without repair; loop electrode excision
58120	1	Dilation and curettage, diagnostic and/or therapeutic (nonobstetrical)
58140	3	Myomectomy, excision of fibroid tumor(s) of uterus, 1 to 4 intramural myoma(s) with total weight of 250 g or less and/or removal of surface myomas; abdominal approach spondylolisthesis
58150	3	Total abdominal hysterectomy (corpus and cervix), with or without removal of tube(s), with or without removal of ovary(s);
58180	3	Supracervical abdominal hysterectomy (subtotal hysterectomy), with or without removal of tube(s), with or without removal of ovary(s)
58260	2	Vaginal hysterectomy, for uterus 250 g or less;
58262	2	Vaginal hysterectomy, for uterus 250 g or less; with removal of tube(s), and/or ovary(s)
58550	3	Laparoscopy, surgical, with vaginal hysterectomy, for uterus 250 g or less;
58552	3	Laparoscopy, surgical, with vaginal hysterectomy, for uterus 250 g or less; with removal of tube(s) and/or ovary(s)
58565	2	Hysteroscopy, surgical; with bilateral fallopian tube cannulation to induce occlusion by placement of permanent implants
58571	3	Laparoscopy, surgical, with total hysterectomy, for uterus 250 g or less; with removal of tube(s) and/or ovary(s)
58720	2	Salpingo-oophorectomy, complete or partial, unilateral or bilateral (separate procedure)
60210	2	Partial thyroid lobectomy, unilateral; with or without isthmusectomy
60220	2	Total thyroid lobectomy, unilateral; with or without isthmusectomy
60240	2	Thyroidectomy, total or complete

© 2020 Shinall MC Jr et al. *JAMA Network Open.*

CPT Code	OSS	CPT Description
60252	2	Thyroidectomy, total or subtotal for malignancy; with limited neck dissection
60254	3	Thyroidectomy, total or subtotal for malignancy; with radical neck dissection
60260	2	Thyroidectomy, removal of all remaining thyroid tissue following previous removal of a portion of thyroid
60271	3	Thyroidectomy, including substernal thyroid; cervical approach
60280	2	Excision of thyroglossal duct cyst or sinus;
60500	2	Parathyroidectomy or exploration of parathyroid(s);
60540	3	Adrenalectomy, partial or complete, or exploration of adrenal gland with or without biopsy, transabdominal, lumbar or dorsal (separate procedure);
60650	3	Laparoscopy, surgical, with adrenalectomy, partial or complete, or exploration of adrenal gland with or without biopsy, transabdominal, lumbar or dorsal
61154	3	Burr hole(s) with evacuation and/or drainage of hematoma, extradural or subdural
61312	4	Craniectomy or craniotomy for evacuation of hematoma, supratentorial; extradural or subdural
61510	3	Craniectomy, trephination, bone flap craniotomy; for excision of brain tumor, supratentorial, except meningioma
61512	3	Craniectomy, trephination, bone flap craniotomy; for excision of meningioma, supratentorial
61518	3	Craniectomy for excision of brain tumor, infratentorial or posterior fossa; except meningioma, cerebellopontine angle tumor, or midline tumor at base of skull
61548	3	Hypophysectomy or excision of pituitary tumor, transnasal or transseptal approach, nonstereotactic
61750	2	Stereotactic biopsy, aspiration, or excision, including burr hole(s), for intracranial lesion;
61867	3	Twist drill, burr hole, craniotomy, or craniectomy with stereotactic implantation of neurostimulator electrode array in subcortical site (eg, thalamus, globus pallidus, subthalamic nucleus, periventricular, periaqueductal gray), with use of intraoperative microelectrode recording; first array
61885	3	Insertion or replacement of cranial neurostimulator pulse generator or receiver, direct or inductive coupling; with connection to a single electrode array
61886	3	Insertion or replacement of cranial neurostimulator pulse generator or receiver, direct or inductive coupling; with connection to 2 or more electrode arrays
62165	3	Neuroendoscopy, intracranial; with excision of pituitary tumor, transnasal or trans-sphenoidal approach
62223	3	Creation of shunt; ventriculo-peritoneal, -pleural, other terminus
62230	3	Replacement or revision of cerebrospinal fluid shunt, obstructed valve, or distal catheter in shunt system
62362	2	Implantation or replacement of device for intrathecal or epidural drug infusion; programmable pump, including preparation of pump, with or without programming
63001	3	Laminectomy with exploration and/or decompression of spinal cord and/or cauda equina, without facetectomy, foraminotomy or discectomy (eg, spinal stenosis), 1 or 2 vertebral segments; cervical
63003	3	Laminectomy with exploration and/or decompression of spinal cord and/or cauda equina, without facetectomy, foraminotomy or discectomy (eg, spinal stenosis), 1 or 2 vertebral segments; thoracic
63005	3	Laminectomy with exploration and/or decompression of spinal cord and/or cauda equina, without facetectomy, foraminotomy or discectomy (eg, spinal stenosis), 1 or 2 vertebral segments; lumbar, except for
63015	3	Laminectomy with exploration and/or decompression of spinal cord and/or cauda equina, without facetectomy, foraminotomy or discectomy (eg, spinal stenosis), more than 2 vertebral segments; cervical
63017	3	Laminectomy with exploration and/or decompression of spinal cord and/or cauda equina, without facetectomy, foraminotomy or discectomy (eg, spinal stenosis), more than 2 vertebral segments; lumbar

© 2020 Shinall MC Jr et al. *JAMA Network Open.*

CPT Code	OSS	CPT Description
63020	2	Laminotomy (hemilaminectomy), with decompression of nerve root(s), including partial facetectomy, foraminotomy and/or excision of herniated intervertebral disc; 1 interspace, cervical
63030	2	Laminotomy (hemilaminectomy), with decompression of nerve root(s), including partial facetectomy, foraminotomy and/or excision of herniated intervertebral disc; 1 interspace, lumbar
63042	3	Laminotomy (hemilaminectomy), with decompression of nerve root(s), including partial facetectomy, foraminotomy and/or excision of herniated intervertebral disc, reexploration, single interspace; lumbar
63045	2	Laminectomy, facetectomy and foraminotomy (unilateral or bilateral with decompression of spinal cord, cauda equina and/or nerve root[s], [eg, spinal or lateral recess stenosis]), single vertebral segment; cervical
63047	2	Laminectomy, facetectomy and foraminotomy (unilateral or bilateral with decompression of spinal cord, cauda equina and/or nerve root[s], [eg, spinal or lateral recess stenosis]), single vertebral segment; lumbar
63050	3	Laminoplasty, cervical, with decompression of the spinal cord, 2 or more vertebral segments;
63051	3	Laminoplasty, cervical, with decompression of the spinal cord, 2 or more vertebral segments; with reconstruction of the posterior bony elements (including the application of bridging bone graft and non-segmental fixation devices [eg, wire, suture, mini-pl
63075	2	Discectomy, anterior, with decompression of spinal cord and/or nerve root(s), including osteophytectomy; cervical, single interspace
63081	3	Vertebral corpectomy (vertebral body resection), partial or complete, anterior approach with decompression of spinal cord and/or nerve root(s); cervical, single segment
63267	3	Laminectomy for excision or evacuation of intraspinal lesion other than neoplasm, extradural; lumbar
63655	2	Laminectomy for implantation of neurostimulator electrodes, plate/paddle, epidural
63685	2	Insertion or replacement of spinal neurostimulator pulse generator or receiver, direct or inductive coupling
63688	2	Revision or removal of implanted spinal neurostimulator pulse generator or receiver
64708	2	Neuroplasty, major peripheral nerve, arm or leg, open; other than specified
64718	2	Neuroplasty and/or transposition; ulnar nerve at elbow
64721	1	Neuroplasty and/or transposition; median nerve at carpal tunnel
64831	2	Suture of digital nerve, hand or foot; 1 nerve
69714	3	Implantation, osseointegrated implant, temporal bone, with percutaneous attachment to external speech processor/cochlear stimulator; without mastoidectomy

© 2020 Shinall MC Jr et al. *JAMA Network Open.*

eFigure: Revised RAI Scoring

RISK ANALYSIS INDEX (RAI)<br>REVISED SURVEY ITEMS[1] & REVISED SCORING SYSTEM[2]			
PATIENT NAME: LAST	FIRST		Middle
DATE OF BIRTH	AGE	GENDER ☐ MALE __3__	☐ FEMALE
MRN:	HEIGHT:	WEIGHT:	
PERSON COMPLETING THIS FORM IF OTHER THAN THE PATIENT:	NAME:	RELATIONSHIP	

Social_History

1) Do you live in a nursing home, skilled nursing facility or another assisted living environment?

☐ No (+0) ☐ Yes (+1)

If yes, Where do you live?

☐ Nursing Home ☐ Skilled Nursing Facility ☐ Assisted Living

If yes, When did you begin living in a nursing home, skilled nursing facility or another assisted living environment?

☐ Less than 3 months ago ☐ 3 months – 1 year ago ☐ Greater than 1 year ago

Medical History

2) Have you ever seen a nephrologist (kidney doctor) or have a history of kidney problems?

☐ No ☐ Yes

If yes, Was this for kidney stones or another problem (choose only 1)?

☐ Kidney Stone (+0) ☐ Other (+8) ☐ Both kidney stones and another problem (+8)

3) Do you have chronic (long-term) congestive heart failure (CHF)?

☐ No (+0) ☐ Yes (+5)

4) Do you have shortness of breath while resting or with minimal activity?

Prompt: Do you use oxygen at home at any time or use oxygen for increased activity?

☐ No (+0) ☐ Yes (+3)

© 2020 Shinall MC Jr et al. *JAMA Network Open.*

5) In the past 5 years, have you been diagnosed or treated for cancer other than basal cell cancer?

☐ No (+0) ☐ Yes (See Chart)

6) In the past 3 months, have you lost 10 pounds or more without trying?

☐ No (+0) ☐ Yes (+4)

7) Is your appetite currently poor?

☐ No (+0) ☐ Yes (+4)

8) During the last 3 months, has it become more difficult for you to remember things or organize your thoughts?

☐ No (+0) ☐ Yes (see chart)

Activities of Daily Living

Mobility:

☐ Can get around without any help. (+0)

☐ Needs help from a cane, walker or scooter. (+1)

☐ Needs help from others to get around the house or neighborhood. (+2)

☐ Needs help getting in or out of a chair. (+3)

☐ Totally dependent on others to get around. (+4)

Eating:

☐ Can plan and prepare own meals. (+0)

☐ Needs help planning meals. (+1)

☐ Needs help preparing meals. (+2)

☐ Needs help eating meals. (+3)

☐ Totally dependent on others to eat meals. (+4)

Toileting:

☐ Can use the toilet without help. (+0)

☐ Needs help getting to or from the toilet. (+1)

☐ Needs help to use toilet paper. (+2)

☐ Cannot use a standard toilet, but with help can use a bedpan/urinal. (+3)

☐ Totally dependent on others to manage toileting. (+4)

Personal Hygiene:

☐ Can shower or bathe without prompting or help. (+0)

☐ Can shower or bathe without help when prompted. (+1)

© 2020 Shinall MC Jr et al. *JAMA Network Open.*

☐ Needs help preparing the tub or shower. (+2)

☐ Needs some help with some elements of washing. (+3)

☐ Totally dependent on others to shower or bathe. (+4)

Total ADL Score=Mobility+Eating+Toileting+Hygiene (range 0-16): ____

Total RAI Score____________ (See chart to calculate Age*Cancer and ADL*Cog scores; then sum all remaining items as scored above)

© 2020 Shinall MC Jr et al. *JAMA Network Open.*

**Variable**	**Revised RAI**	
Sex	3	
Age*Cancer	w/o cancer	w/ cancer
Age		
<=19	0	28
20-24	1	29
25-29	4	29
30-34	6	30
35-39	8	30
40-44	10	31
45-49	12	31
50-54	14	32
55-59	16	32
60-64	18	33
65-69	20	34
70-74	22	34
75-79	24	35
80-84	26	35
85-89	28	36
90-94	30	36
95-99	32	37
100+	34	37
Weight Loss	4	
Poor Appetite	4	
Renal Failure	8	
Chronic/Congestive Heart Failure	5	
Shortness of Breath	3	
Residence other than Ind. Living	1	
ADL*Cog	w/o cog	w/ cog
ADL Score		
0	0	5
1	1	6
2	2	6
3	3	7
4	4	8
5	4	8
6	5	9
7	6	10
8	7	11
9	8	11
10	9	12
11	10	13
12	11	13
13	11	14
14	12	15
15	13	15
16	14	16
Total RAI	0	81

© 2020 Shinall MC Jr et al. *JAMA Network Open.*

eReferences

[1]Varley PR, Borrebach JD, Arya S, Massarweh NN, Bilderback AL, Wisniewski MK, Nelson JB, Johnson JT, Johanning JM, Hall DE. Clinical Utility of the Risk Analysis Index as a Prospective Frailty Screening Tool within a Multi-practice, Multi-hospital Integrated Healthcare System. *Ann Surg. 10.1097/SLA.0000000000003808.* 2020

[2] Arya S, Varley P, Youk A, Borrebach JA, Perez S, Massarweh NN, Johanning JM, Hall DE. Recalibration and External Validation of the Risk Analysis Index: A Surgical Frailty Assessment Tool. *Ann Surg. 10.1097/SLA.0000000000003276.* 2019.

© 2020 Shinall MC Jr et al. *JAMA Network Open.*
